# Influence of the Gut Microbiota on the Pathogenesis of Alzheimer’s Disease: A Literature Review

**DOI:** 10.3390/cells14201578

**Published:** 2025-10-11

**Authors:** Joanna Koga-Batko, Katarzyna Antosz-Popiołek, Wojciech Suchecki, Hubert Szyller, Martyna Wrześniewska, Maciej Dyda, Jerzy Leszek

**Affiliations:** 1 Faculty of Medicine, Wroclaw Medical University, Ludwika Pasteura 1, 50-367 Wrocław, Poland; antosz0715@gmail.com (K.A.-P.); wsuchecki881@gmail.com (W.S.); hermeszyller@gmail.com (H.S.); martyna.wrzesniewska@gmail.com (M.W.); macdyda@gmail.com (M.D.); 2 Clinic of Psychiatry, Department of Psychiatry, Wroclaw Medical University, Ludwika Pasteura 10, 50-367 Wrocław, Poland

**Keywords:** Alzheimer’s disease, gut microbiota, gut–brain axis, lipopolysaccharides, short-chain fatty acids, neuroinflammation, probiotics, prebiotics

## Abstract

Alzheimer’s disease (AD) is a progressive neurodegenerative disorder with a complex etiology whose exact mechanisms are not fully understood. In recent years, there has been growing interest in the role of the gastrointestinal microbiota in the pathogenesis of AD, particularly in the context of the gut–brain axis. The purpose of this review is to discuss the current state of knowledge regarding potential relationships between the composition of the gut microbiota and the development and progression of AD. Preclinical and clinical studies indicating that microbiota imbalances (dysbiosis) may contribute to increased inflammation, increased permeability of the intestinal and blood–brain barriers, and accumulation of pathological proteins such as beta-amyloid and tau are analyzed. The effects of diet, probiotics and microbiota interventions on cognitive function were also discussed. An attempt was also made to evaluate microbiota disruption as a potential early marker of AD development. Although the mechanisms require further study, the role of the gut microbiota appears to be an important and promising direction in understanding the pathophysiology of AD and developing potential therapeutic and diagnostic strategies.

## 1. Introduction

Alzheimer’s disease (AD) is the most common cause of dementia worldwide. It is estimated that the global number of patients will increase from 55 million in 2021 to 139 million in 2050 [1]. It is a progressive neurodegenerative disease. When analyzing its pathomechanism, special emphasis is placed on beta amyloid deposition and hyperphosphorylation of tau protein [2,3]. Interest in the role of chronic neuroinflammation and its impact on the genesis, course and dynamics of Alzheimer’s disease has increased significantly. Many authors view the chronic inflammatory process of the CNS as the main mechanism of AD pathogenesis, linking the other pathways in the development of the disease [4,5]. It is a complex relationship involving cells: min. astroglia, microglia and proteins such as cytokines and chemokines. This results in neuronal damage, cell apoptosis, and ultimately the development of dementia symptoms [6].

The brain, along with the gut, is connected by a very strong bidirectional communication network termed the gut–brain axis. It consists of immunological, endocrine, neuronal, and metabolic mechanisms [7]. One component of this interaction is the microbiota. It is estimated that our gut is inhabited by more than 1000 species and about 7000 strains of bacteria [8]. The microbiota, in turn, is defined as the collection of microorganisms such as bacteria, fungi, and viruses that inhabit the gastrointestinal tract. Over the past few years, the role of the gut microbiota in triggering neuroinflammation has attracted particular attention from researchers. Dysbiosis, i.e., its disruption of composition and diversity, via metabolites, neurotransmitters and inflammatory factors, influences the development of AD [9]. In addition, dysbiosis translates into increased permeability of the intestinal barrier and the blood–brain barrier, which allows substances such as lipopolysaccharides, short-chain fatty acids and bacterial amyloids to flow into the blood and then to the CNS, where they induce inflammation [8].

Today, the scientific community is increasingly in favor of recognizing Alzheimer’s Disease as a systemic disease, the treatment of which will target a variety of causes of cognitive impairment. One of the targets for potential therapy is the gastrointestinal flora. Despite promising preclinical studies, the availability of clinical data is still significantly limited [10,11]. Therefore, further research is needed to reliably evaluate the effects of probiotic, prebiotic and microbiota transplantation (FMT) therapies in both the treatment and prevention of Alzheimer’s disease. The role antibiotics play in the pathogenesis of AD, mediated by modulation of the microbiota, is ambiguous. Animal models suggest that long-term antibiotic therapy, by inducing dysbiosis, can increase interleukin levels in the brain, leading to neuroinflammation and reduced quality of cognitive memory [12]. In contrast, elimination of chronic H. pylori infection can result in improved cognitive function [13]. Nevertheless, antibiotics should be one avenue for exploring potentially new methods of AD prevention and treatment.

The review brings together the latest information on the complex, reciprocal interactions between the gastrointestinal bacterial flora and the pathogenesis of Alzheimer’s disease. In addition to analyzing the effects of dysbiosis, the potential hopes raised by modifying the composition of the bacterial flora in improving patients’ cognitive function, as well as in the prevention of the disease itself, will be discussed.

## 2. Alzheimer’s Disease—Basic Facts, Etiopathology, Symptoms, Diagnosis

Alzheimer’s disease is a progressive neurodegenerative disease of the central nervous system responsible for 60–80% of cases of dementia. In the US, it was the seventh leading cause of death in 2022 and the fifth leading cause of death in the over-65 age group [14,15,16,17], while an estimated 7.2 million US citizens over the age of 65 suffer from AD, and this number is projected to reach 13.8 million by 2060, given the limited therapeutic options available and the aging population [18]. Given the increasing incidence and detection rates of Alzheimer’s disease, it poses an increasingly serious challenge to public health care worldwide, contributing not only to systemic but also financial burdens [16,17,19,20].

The pathomechanism responsible for the development of this type of dementia remains not fully understood. Progressive memory loss and cognitive impairment are commonly associated with the accumulation of amyloid-beta oligomer plaques in the extracellular space, which have a toxic effect on neurons and disrupt transmission between synapses. Hyperphosphorylated tau protein is also considered a possible etiopathological factor, whose intracortical accumulation causes dysfunction of the neuron’s microtubule system, thus disrupting the intracellular transport of nutrients and cellular metabolites [21,22]. Protein accumulation also leads to pathological atrophy of the nucleus basalis of Meynert by stimulating the activity of pro-inflammatory cytokines, resulting in chronic inflammation [23,24]. Another factor contributing to the development of Alzheimer’s disease is increasingly recognized as blood–brain barrier dysfunction resulting from age-related vascular stress, whose compromised selectivity prevents the effective transport of beta-amyloid to the peripheral circulation, thereby increasing the rate of protein accumulation and, consequently, its pro-inflammatory effect [25]. Among the conditions contributing to Blood–Brain Barrier (BBB) dysfunction is hypercholesterolemia, which is common among the elderly [26].

The clinical picture of Alzheimer’s disease includes a wide range of symptoms, both less and more characteristic, which also fluctuate in severity during the course of the disease [18]. First, there is general distraction, isolated losses in vocabulary, and memory impairment. Over time, these are accompanied by behavioral changes, mood swings, and previously unseen behaviors such as wandering without a specific purpose. One of the most commonly observed initial symptoms is a personality change; the patient may become unpleasant to loved ones, aggressive, and self-centered. After some time, motor disorders appear in movement and basic activities of daily living, such as swallowing. The average life expectancy after diagnosis is estimated at 4 to 8 years [16,17,18]. There are many risk factors for developing Alzheimer’s disease. The most important ones include:

- demographic factors: age, gender, education,
- genetic factors: *APOE-e4*, genetic mutations in the *APP*, *presenilin-1 and presenilin-2* genes, Down syndrome, family history among first-degree relatives,
- lifestyle: smoking, physical inactivity, obesity, lack of intellectual stimulation, malnutrition, poor diet,
- clinical condition: cancer, cardiovascular disease, metabolic syndrome, and hypercholesterolemia
- as well as environmental factors, psychiatric disorders, and even infections [17,18,27].

The diagnosis of Alzheimer’s disease is varied and includes laboratory, psychiatric, and radiological components. The DSM-5 criteria indicate that memory and cognitive decline should be considered suspicious for Alzheimer’s disease when accompanied by adverse genetic results or if the disorders are progressive and no mixed etiology is suspected [28]. Laboratory diagnostics use blood measurements of amyloid beta and tau protein levels, which help to distinguish dementia of other etiologies from Alzheimer’s disease, but the required equipment significantly limits its use in clinical practice. Elevated levels of pro-inflammatory factors such as interleukin-6, 12, and 18, as well as tumor necrosis factor (TNF) and transforming growth factor (TGF), also allow for earlier detection of the disease. An additional diagnostic step is imaging tests: CT, MRI, and PET. These allow monitoring of disease progression in the form of progressive atrophy in areas responsible for memory and emotions, such as the hippocampus and occipital and parietal cortex [16,21].

Currently, with an increasingly better understanding of the mechanisms of the microbiota-gut–brain axis, many studies point to a significant link between neurodegenerative diseases, such as Alzheimer’s disease, and the effectiveness of diagnosis and optimization of therapeutic management [9].

## 3. Gut–Brain Axis in AD

To properly understand the issues discussed, it seems necessary to present the gut–brain axis itself. When talking about this axis, it is important to note that it is always considered bidirectional. There is mutual communication between the central nervous system (CNS) and the gastrointestinal tract. The intestines are equipped with the gut microbiota and the enteric nervous system (ENS) itself, which is sometimes called the “second brain”. Signaling molecules produced by gut microbes interact with the CNS. This communication seems to occur at all stages of development, at different points in time, and through different modes of communication such as neural, immune, endocrine, and metabolic signaling [29,30,31]. It is important to mention, the ENS can act independently, modulate, and react on its own, but it also constantly has a connection and mutual interactions with the CNS. This connection, although it has many advantages, also allows the spread of diseases and the transfer of pathogenic mediators further [32].

The bacteria found in the digestive system, the gut microbiota, are mostly harmless or beneficial to humans. Only a few species can cause disease or pose any risk, and even if some bacteria are naturally dangerous, the intestinal microbiota works on the principle of balance, where the action of some species can be balanced by others. Only disturbances of the flora, called *dysbiosis*, with the dominance of some species over others, can cause disorders [33].

### 3.1. Microbiota in AD

The composition of the microbiota is established at the beginning of life, and in healthy individuals, it is relatively similar throughout most of life. With age and the emergence of chronic diseases, toxins, and medications, this composition may be disturbed, and as a result, the older patients are in a chronic low-grade state of inflammation [29], which will be described further.

It is currently estimated that there may be over a thousand species, or in other words, seven thousand strains of bacteria in the human intestines [8,34]. A significant part of the human microbiota, that is up to eighty percent, consists of the phyla *Bacteroidetes* and *Firmicutes.* Other phyla worth noting are *Actinobacteria*, *Fusobacteria*, and *Verrucomicrobia*. Typically pathogenic bacteria, such as *Campylobacter jejuni*, *Salmonella enterica*, *Vibrio cholerae*, *Escherichia coli*, or *Bacteroides fragilis*, are usually around 0.1% [33,35,36].

Referring to the largest groups, the following indicator was proposed—the Firmicutes/Bacteroidetes (F/B) ratio. This ratio between these two phyla is already a documented indicator for diseases, e.g., obesity [37]. Studies described by, among others, Cattaneo et al. and Vogt et al. proved the existence of disproportions in the percentage of microflora. Samples taken from AD patients contained a reduced percentage of bacteria acting beneficially, while an increase in pathological bacteria was noticeable [38,39]. The proportion of the two phyla was also imbalanced, with a reduced level of *Firmicutes*, causing the dysbiosis.

It should be emphasized, however, that this does not mean directly calling individual types “good” or “bad”, because the properties of the microbiome are determined by the proportions, diversity, and stability of the resulting network of dependencies [9,40].

### 3.2. Firmicutes

Special attention should be paid to *Firmicutes,* the largest bacterial phylum in human microbiota. These bacteria are mostly Gram-positive with a thick peptidoglycan layer. *Firmicutes* have over 200 genera, such as *Ruminococcus*, *Clostridium*, *Eubacterium*, *Lactobacillus*, *Faecalibacterium*, *Roseburia*, and *Mycoplasma* [33,40]. A significant feature of these bacteria is the fermentation of indigestible carbohydrates (such as dietary fiber) and the production of short-chain fatty acids (SCFAs). SCFAs are a crucial part of the intestinal barrier, as they provide nutrients to epithelial cells, and can regulate as well as help the human immune system. In several studies from 2017 to 2021, a disproportion between microbiota producing SCFAs from healthy and AD patients was described. Research shows that AD patients experience a decrease in SCFA-producing species, such as *Lachnospira*, *Ruminoclostridium 9*, *Clostridiaceae*, *Lachnospiraceae*, and *Ruminococcus*. Additionally, it is worth adding that these varieties were also responsible for better results in various types of tests checking cognitive skills [38,40,41,42,43,44].

Staying on the subject of securing the digestive tract, one compound from SCFAs in particular deserves a broader discussion—butyrate. It is a desired compound by the colonic epithelial cells, for which it is a source of energy. Moreover, butyrate has anti-inflammatory properties and modulates the immune system [45], and here again, the same situation, patients with AD seem to have less numerous species such as *Eubacterium rectale*, *Eubacterium eligens*, and *Eubacterium halli* [42]. Referring to the flagship hotspot of AD pathogenesis, Aβ aggregation seems to be connected with butyrate production. Studies prove that in Aβ-positive patients, in comparison to Aβ-negative ones, a decrease in *E. rectale* species is observed [39].

Another species to be described is *Lactobacillus*, as it also produces valuable particles, e.g., gamma-aminobutyric acid (GABA) and acetylcholine. These structures play a significant role in neural communication, propagating specific information in the system. There are more and more studies on the use of therapy using probiotics to improve cognitive functions [46]. However, a paragraph focused directly on the applications of these studies, on the sublimation of probiotics, and the possibilities of therapy will be presented in a later part of the publication.

### 3.3. Bacteroidetes

When it comes to *Bacteroidetes*, these bacteria have a Gram-negative cell wall structure, with an outer membrane built from lipopolysaccharide (LPS), which in turn is associated with the host’s immune responses. These bacteria include the genera *Bacteroides, Prevotella*, and *Xylanibacter* [33,40]. Many species within this phylum stimulate intestinal function, produce valuable metabolic compounds, and support the immune system by preventing colonization of hostile pathogens [40]. Although there are also species that produce enterotoxins that disrupt the intestinal barrier. Studies also indicate a dangerous phenomenon where LPS, crossing the intestinal barrier, not only causes endotoxemia but also accumulates together with Aβ plaques in the brain [47]. If this condition is prolonged and exposure to LPS is constant, production and aggregation of Aβ progress noticeably. Moreover, there are studies suggesting a protective function of Aβ against microorganisms such as bacteria or fungi [29,48,49]. The dual phenomenon of protection and destruction of the molecule described here is not an isolated phenomenon in biological pathways; similar relationships occur in the case of other antimicrobial peptides. It is also worth noting that the studies on this phylum are not so clear. The studied relationships in the amount of this microflora in patients with AD and healthy patients are not consistent; the values are sometimes increased in some studies and sometimes decreased [38,39,41,42,44].

To summarize the above two paragraphs, a table with the most important information is presented.

### 3.4. The Inflammatory Process on the Gut–Brain Axis

The already mentioned dysbiosis contributes to the overall proinflammatory effect. The disturbance of microbiological balance affects the emerging inflammatory metabolites, leading to a series of biochemical reactions that ultimately also affect the neuroinflammation process. This comes down to the worsening of the formation and accumulation of Aβ, already described in the pathogenesis of AD.

Bacteria can be divided into those with a resultant pro- or anti-inflammatory effect. This division is determined by the relationship on the gut–brain axis, but also generally, as the metabolites produced by bacteria, which are sometimes toxins, get entangled in the biochemical pathways of the whole human body [8]. In the following part of the work, there is also a table (Table 1) summarizing several such properties of the resultant bacteria. However, it is also worth taking a closer look at them.

Studies of this type are based, for example, on measuring the number of specific bacteria in the patient’s stool/ transgenic mice and the level of expression of pro-inflammatory (e.g., interleukins (IL-6, IL-18, IL-8), inflammasome complex (NLRP3), tumor necrosis factor-alpha (TNF-α)) and anti-inflammatory cytokines (e.g., IL-4, IL-10, IL-13). Such results are applied to patients with AD and healthy individuals in search of relationships [38,39,44]. And so, for example, in research conducted by Cattaneo et al., cognitively impaired patients indicate higher levels of proinflammatory cytokines (IL-6, CXCL2, NLRP3, and IL-1β). Additionally, the reduction in anti-inflammatory cytokine IL-10 was observed in this group. Their patients showed lower abundance of *E. rectale* and higher abundance of Escherichia/Shigella compared with both healthy controls [39].

The beneficial role of *Firmicutes* in the production of SCFAs has already been described earlier as an example of an anti-inflammatory effect. On the other hand, a relationship of polyunsaturated fatty acids (PUFA) and the gut microbiota with *Bacteroides* enrichment collected from AD patients was described. These bacteria seem to activate a pro-inflammatory PUFA cascade in the brain. The resulting PUFAs appear to stimulate microglial maturation in the brain, pathological processes associated with AD, and cognitive deficits [50].

With the growing interest in this topic, more and more studies have been conducted around this content, some based on mouse models, and some on the human microbiome. Based on the work available in the databases, a summary of several bacteria and their resulting action in the gut–brain axis is presented, with the focus on the type of inflammatory response (Table 2).

Remaining on the topic of the intestines, the bacteria that live there give their surroundings signs of inflammation, which in turn is related to macrophage dysfunction and the so-called “leaky gut” hypothesis. The resulting systemic inflammation affects the already known and well-presented above the BBB. Overall, a predominance of pro-inflammatory microbiome contributes to increased intestinal permeability, which in turn affects the strength and integrity of the BBB, contributing to the development of disorders occurring in AD [29,53].

### 3.5. A Different Approach to the Topic

Finally, it is also worth noting another idea describing the relationship between AD and microbiota. Some studies and theories consider the direct process of bacterial infection as a causative factor. In these connections, researchers refer to typical infectious phenomena and the possibility of spreading the pathogenic process after entering the bloodstream. Such phenomena are known, for example, in the case of viruses (particularly HSV-1, EBV, and HCMV). However, it is unclear if these events simply coexist in time without such a connection. It is difficult to prove a finding of a specific infection that could cause a disease as slowly developing as AD, with amyloid deposition that begins 15–20 years before symptoms appear [54,55].

The theory of the direct influence of microorganisms is already 30 years old. However, every few years, the potential value of this theory is regained attention again, as, among others, thanks to the discovery of antimicrobial properties of Aβ against bacteria and fungi [49]. As an example, it is worth mentioning the study conducted using the HSV-1 virus, where the Aβ oligomers seem to inhibit HSV-1 infection in vitro. This study could suggest that the encapsulation of the brain tissue with Aβ aggregates is initially a protective function before it turns into a pathogenic process through malfunctioning repair mechanisms. Although this remains speculative and requires further mechanistic validation in a clinical context [56]. These studies definitely require more recent research with clinical trials, but it is worth being aware of them. Finally, it is worth mentioning that the bacteria in the intestines are themselves a huge source of amyloids. In bacteria, e.g., *E. coli*, it has a protective function and helps build a biofilm that wards off the immune system’s response. The structure of bacterial amyloids is indeed different from human amyloids, but there is a noticeable similarity in their tertiary structure, which triggers a similar response of the immune system to both of these formats [57,58]. Additionally, bacterial amyloids can act through molecular mimicry and affect host proteins, causing pathological cross-reactions, changing the structures of other proteins [59].

To sum this all up, with more and more information and awareness of all the connections, it seems that the right answer to the link between AD and human microbiota cannot be a single finding. It is probably the result of many individual processes, including both inflammatory processes and the direct impact of microorganisms, overlapping and interacting, some with greater force, others with less.

## 4. Antibiotics

Assuming the influence of the gut microbiota on the development and progression of Alzheimer’s disease, it is impossible to ignore the impact of substances that significantly alter the structure of the microbiome, such as antibiotics. Given the wide spectrum of bacteria eliminated by antibiotic therapy, the gut microbiota is reduced, and as a result of its reduced diversity, the risk of dysbiosis increases, and a long-term process of spontaneous restoration is necessary to fill the niche, which is susceptible to occupation by pathogenic strains. This has both short-term and long-term consequences [13,60,61].

Current knowledge about the impact of antibiotics on Alzheimer’s disease reveals a complex relationship between the microbiota and the development of neurodegenerative diseases [62].

Much evidence, mainly based on animal studies and preclinical models, points to the positive effect of antibiotic therapy on inhibiting the progression of AD. Studies in mice have shown that numerous and long-lasting changes in the microbiota, achieved with a wide range of antibiotics, led to a reduction in accumulated Aβ and an increase in soluble Aβ, with simultaneous attenuated plaque-localised glial reactivity [62].

Research is also being conducted on the use of minocycline and doxycycline, which have anti-aggregatory properties against Tau and Aβ proteins, while also exhibiting anti-inflammatory activity [63]. Studies conducted on animal models confirm these properties, but point to numerous complications resulting from chronic use of tetracyclines [64].

Rifampicin also exhibits similar anti-aggregatory, brain-protective properties [65]. In animal model studies, it enhanced autophagy in hippocampal cells, thereby reducing Aβ accumulation [66].

Sirolimus (formerly Rapamycin), in short-term administration in animal models, caused restoration of the Brain Volumes of the Hippocampus and Caudate, which play an important role in cognitive abilities, memory formation and Global Cerebral Blood Flow among APOE4 carriers [67]. Rapamycin is an inhibitor of the mammalian enzyme target of rapamycin (mTOR), whose regulation plays an important role in the pathogenesis of Alzheimer’s disease [13]. In the case of beta-lactam antibiotics, preclinical studies suggest their effectiveness in inhibiting the progression of AD [68].

In the Korean cohort study conducted by Kim et al., published in 2022, it has been suggested that antibiotic therapy may contribute to the development of chronic inflammation in the nervous system and exacerbate the course of Alzheimer’s disease [60]. It has been shown that dysbiosis caused by quinolone consumption may contribute to an increased risk of developing AD, possibly due to different short-chain fatty acids and lipopolysaccharides produced by the metabolism of the intestinal flora altered by antibiotics [69]. A cohort study of the Korean population showed a risk of developing dementia in a cumulative duration-dependent manner among adults [60]. Furthermore, antibiotics administered as a multi-component cocktail may transiently impair cognition, induce neurobehavioral symptoms, and disrupt gut–brain axis homeostasis [13].

Table 3 summarizes information about antibiotics presented above.

## 5. Potential Biomarkers of AD Based on Gut-Microbiota

When it comes to the diagnosis of AD, there is an urgent need to identify biomarkers that could enable its early detection. As gut microbiota has been found to play a role in neurodegenerative diseases, it is believed that biomarkers based on it could be an alternative in the diagnostic process.

There have been several studies on APP/PS1 transgenic mice that have proposed some new potential biomarkers. It was found that in APP/PS1 transgenic mice, the number of SCFA-producing bacteria, *Bacteroidaceae* and *Rikenellaceae* was lower than in controls, and on the other hand, the number of *Firmicutes*, *Bacteroidetes*, *Proteobacteriaceae*, *Verrucomicrobiaceae*, *Bifidobacteriaceae*, *Erysipelotrichaceae*, *Helicobacteraceae* and *Desulfovibrionaceae* was higher [70,71,72,73]. It was also found that those changes in the microbiome precede the deposition of plaques, which makes them promising early markers [73]. Studies on humans were also conducted, and it was found that there are higher levels of Trimethylamine N-oxide (TMAO) in the cerebrospinal fluid in patients with AD than in healthy controls [74]. Other potential biomarkers include short-chain fatty acids and tryptophan metabolites, which modulate neuroinflammation and maintain BBB integrity, such as indole-3-pyruvic acid, whose levels are lower in patients with AD [75]. Studies also found that individuals with AD have lower microbial diversity, and in their gut microbiota, there is a predominance of butyrate-producing bacteria over lactate-producing bacteria [76] [Table 4].

This data seems promising; however, there is still a need for validation of those potential markers in studies including larger groups of patients and healthy controls, especially because the gut microbiome is influenced by many factors, such as dietary habits or lifestyle [77]. Thus, the possible applications of microbiota changes in patients with AD in the diagnostic process are an interesting and necessary topic for scientists to explore.

## 6. The Potential of Prebiotics, Probiotics, and Dietary Interventions in the Prevention and Therapy of Alzheimer’s Disease

While the impact of dietary patterns on AD progression had been a topic of numerous studies, nowadays, more researchers focus on how microbiome alterations in AD patients influence the disease progression and how they can delay AD and related dementia (ADRD) development.

### 6.1. Diet

Studies have shown a causal relationship between the Western diet and pathological brain aging, with evidence of poorer cognitive function among older adults [78]. The Western diet, characterized by high intake of refined products (grains, sugars, saturated fats, salt, and highly processed foods) as well as extremely low fruit and vegetable consumption, resulting in very low fiber levels, has been linked to disturbances in the gut microbiome, resulting in immune dysfunction and inflammation [79,80]. For example, a Western diet rich in saturated fats (SFA) can induce the development of inflammation. SFA triggers TLR4 through CD14/MD2 activation, thereby promoting the expression of the transcription factor NF-κB, which plays a key role in the induction of pro-inflammatory mediators COX2, TNFα, IL-1β, IL-6, CXCL8, IL-12, and IFNγ. Western dietary patterns also promote hypercholesterolemia and insulin resistance, which impair blood flow in the brain and are associated with the development of both Alzheimer’s disease and ADRD [81].

The Mediterranean diet (MeDi), comprising fresh vegetables, fruits, fish, legumes, cereals, and virgin olive oil, has been widely studied for its cognitive benefits. Early studies found that individuals with higher adherence to the Mediterranean diet have a lower likelihood of developing Alzheimer’s disease over a follow-up period of four years, and MeDi is connected to lower mortality among AD patients [82,83]. Furthermore, a systematic review and meta-analysis by Hill et al. found that adherence to a Mediterranean-style diet was correlated with a decrease in levels of AD-related biomarkers, including β-amyloid and tau tangles [84]. Oleuropein, an antioxidant component of olive oil, has been shown to aid in the cleavage of amyloid precursor protein, a precursor of Aβ, and the aglycone compound of oleuropein has therapeutic potential in AD pathology [85].

The benefits of MeDi are a consequence of the components found in its staple products, which are summarized in Table 5.

The benefits of MeDi may be due to the components contained in its key products. Extra virgin olive oil (EVOO), particularly rich in phenolic compounds, has strong antioxidant properties [86]. EVOO metabolites are involved in microbial immunomodulation—a study by Martín-Peláez et al. showed that EVOO consumption stimulates the immune system (measured, among other things, by the level of IgA-coated bacteria in feces and CRP in plasma) [87]. EVOO consumption also enhanced the anti-inflammatory effect of the diet by reducing markers of inflammation and oxidative stress (decrease in MPO, 8-OHdG, TNF-α, and IL-6) and increasing the level of adiponectin, a protein that regulates the expression of the IL-10 gene, which supports the production of anti-inflammatory cytokines [89].

High intake of soluble fiber, abundant in MeDi products, is associated with a lower risk of AD in older adults. Fiber regulates the production of SCFAs by the gut microbiota. In a study by Cuervo-Zanatta et al., increased soluble fiber intake in APP/PS1 transgenic mice restored the balance of the gut microbiota, lowering propionate levels and increasing butyrate production, which led to reduced astrocyte activation due to increased expression of neurotrophic factors (BDNF, GDNF) and decreased expression of A1 markers (C3, Serping1). Butyrate supports the transition to the A2 phenotype—anti-inflammatory microglia [89,90].

Patients following a Mediterranean diet were found to have a greater abundance of species, particularly the Prevotella enterotype [91,92]. A study by Gutiérrez-Díaz et al. showed that MeDi increased the amount of bacteria from the Bacteroidetes, Prevotellaceae, and Prevotella groups and decreased the amount of Firmicutes and Lachnospiraceae [93].Another study showed that people following the MeDi diet had increased levels of Clostridium XIVa and Faecalibacterium prausnitzii, a known producer of anti-inflammatory butyrate: it inhibits NF-κB activation, thereby reducing the production of inflammatory cytokines, inhibits HDAC, affects the epigenetic silencing of inflammatory genes, and protects against the translocation of bacteria and toxins (e.g., LPS) into the blood, which could cause systemic inflammation [94,95,96,97].

It has been suggested that modified versions of the MeDi diet may be more effective than direct supplementation. The MIND (Mediterranean-DASH Intervention for Neurodegenerative Delay) diet, which combines elements of MeDi and DASH (Dietary Approaches to Stop Hypertension), shows even stronger links to reducing cognitive decline [98]. Based on similar components as MeDi, the MIND diet additionally emphasizes regular consumption of leafy vegetables, nuts, and berries due to their neuroprotective properties [99]. A prospective study of the MIND diet conducted by Morris et al. showed that high adherence to this diet for 4.5 years could reduce the risk of developing AD by as much as 53% [100]. However, some studies do not highlight significant benefits of this diet in preventing neurodegeneration compared to calorie restriction alone, so further research is needed [101].

### 6.2. Probiotics

One possible approach to modulating brain function and providing neuroprotection against the onset and progression of Alzheimer’s disease is to directly influence the gut microbiome and its immunomodulatory activity through the use of probiotics. Probiotics, i.e., live microorganisms that have a direct beneficial effect on health, have gained interest in recent years as potential therapeutic agents in the treatment of AD, with particular emphasis on strains of the genus Lactobacillus and Bifidobacterium.

In a study by Bonfili et al., administration of SLAB51 (a probiotic formulation consisting of nine live bacterial strains: *Streptococcus thermophilus*, *Bifidobacterium longum*, *B. breve*, *B. infantis*, *Lactobacillus acidophilus*, *L. plantarum*, *L. paracasei*, *L. delbrueckii* subsp. *bulgaricus*, *L. brevis*) to mice with early-stage AD resulted in a partial improvement in the functioning of two disturbed mechanisms of neuronal proteostasis: the ubiquitin-proteasome system and autophagy, among others, through the activation of sirtuin-1 (SIRT-1)—a neuroprotective deacetylase enzyme that reduces ROS in the CNS [15].

Similar conclusions were reached by Kobayashi et al. (using the Bifidobacterium breve A1 strain in mice with AD) [102] and Medeiros et al., who supplemented transgenic 3xTg-AD mice with a cocktail containing Lactobacillus plantarum KY1032 and Lactobacillus curvatus HY7601 [103]. Probiotic intake led to a significant increase in the number of Bacteroidetes bacteria in the intestinal microflora, a decrease in astrocyte and microglia density in the entorhinal cortex and hippocampus, and an increase in the number of neurons in the hippocampus, indicating a neuroprotective effect [103].

Probiotics may also contribute to slowing the progression of cognitive impairment and Alzheimer’s disease pathology by strengthening the integrity of the gut and BBB while alleviating inflammation in the gastrointestinal tract, systemic circulation, and central nervous system. A decrease in plasma inflammatory markers such as IL-6 and TNF-α has been observed in individuals treated with a probiotic cocktail, while mediators such as TNF-α are responsible for suppressing the expression of tight junction proteins in the brain and intestine, hence greater integrity of, among others, the BBB [104].

### 6.3. Prebiotics

Prebiotics, a group of beneficial nutrients broken down by the gut microbiota, have been the subject of growing interest in recent years as potential factors in preventing Alzheimer’s disease (AD) and delaying its progression [105]. The most commonly used prebiotics include fructooligosaccharides (FOS), galactooligosaccharides (GOS), and trans-galactooligosaccharides (TOS), and their fermentation by gut bacteria leads to the formation of SCFAs, such as lactic, butyric, and propionic acids [106].

FOS are a frequent subject of research due to their promising therapeutic potential in AD via the gut–brain axis. In a study by Sun et al., six weeks of oral administration of FOS to APP/PSEN1dE9 transgenic mice improved cognitive function and reduced AD-related pathological changes by increasing the expression of synapsin I and postsynaptic density protein 95 (PSD-95). In addition, FOS treatment increased GLP-1 levels and decreased the expression of its receptor GLP-1R, thereby modulating the gut microbiota-GLP-1/GLP-1R signaling pathway [107]. In another study, FOS not only improved oxidative stress and inflammation markers but also regulated neurotransmitter synthesis and secretion. Histological analysis also showed reduced neuronal apoptosis and decreased levels of key AD markers such as Tau and Aβ1-42 [108].

GOS, oligosaccharides, also exhibit neuroprotective effects. In a study by de Paiva et al., administration of FOS and GOS to mice on a high-fat diet led to a reduction in neuroinflammation and neuronal damage (as demonstrated by lower levels of TNF-α, COX-2, caspase-3, Iba-1 cells, and GFAP). The treatment improved synaptic plasticity, restored learning and memory abilities, and supported the insulin signaling pathway through activation of the IRS/PI3K/AKT pathway. In addition, it reduced amyloid β formation and Tau phosphorylation, helped restore the balance of the gut microbiota—particularly by increasing the abundance of *Bacteroidetes* bacteria—and reduced intestinal inflammation and permeability [109].

Xylooligosaccharides (XOS) are indigestible oligosaccharides with high prebiotic potential [110]. They strongly stimulate the growth and diversity of *Bifidobactera* in the gut [111]. XOS supplementation also selectively promotes the expansion of strains *Bifidobacterium adolescentis*, known for its involvement in modulating neuroinflammatory pathways [112].

Increasing the diversity of the intestinal microflora through XOS supplementation reduced intestinal inflammation by lowering the levels of pro-inflammatory cytokines IL-1β and IL-6 and immunoregulatory IL-10. Additionally, it increased the expression of tight junction proteins in the intestine, contributing to the restoration of both the epithelial barrier and the blood–brain barrier [113].

Probiotics and prebiotics appear to be promising therapeutic agents for the treatment of AD; however, the currently available scientific evidence comes mainly from studies in animal models. Therefore, further clinical studies involving humans are necessary to confirm their efficacy and practical applicability.

## 7. Conclusions

Previous studies clearly indicate the important role of gut microbiota in the pathogenesis of Alzheimer’s disease (AD). Patients with AD show a marked reduction in the diversity of their gut flora and a decrease in the number of Firmicutes and Bifidobacterium bacteria, accompanied by an increase in Bacteroidetes, Proteobacteria, and pro-inflammatory bacteria such as Escherichia/Shigella. This imbalance, known as dysbiosis, leads to local inflammation in the gastrointestinal tract.

Dysbiosis results in reduced levels of butyrate (butanoic acid)—a short-chain fatty acid with anti-inflammatory and neuroprotective properties—and increased production of reactive oxygen species (ROS). This leads to weakened connections between enterocytes and impaired intestinal barrier integrity. Increased permeability of this barrier, as well as the blood–brain barrier, allows bacterial metabolites and endotoxins to enter the bloodstream and central nervous system (CNS).

Lipopolysaccharides are particularly harmful bacterial products that activate microglia—immune cells in the brain—leading to chronic inflammation in the CNS. In addition, some microbial metabolites affect the activity of the γ-secretase enzyme and, due to their structural similarity to amyloid proteins, can induce abnormal protein folding in a mechanism resembling prion activity. These processes contribute to the development of amyloidogenesis and tauopathy, two key pathologies in Alzheimer’s disease, and exacerbate neuronal dysfunction and cognitive decline.

There are also studies conducted in humans that have shown higher concentrations of trimethylamine oxide (TMAO—a product of choline and L-carnitine metabolism by intestinal bacteria) in the cerebrospinal fluid of AD patients compared to healthy individuals. Other potential biomarkers include SCFAs and tryptophan metabolites such as indole-3-pyruvic acid, which are reduced in AD patients.

Research suggests that early changes in the microbiota may serve as preclinical biomarkers of Alzheimer’s disease. However, the diagnostic potential of these indicators needs to be confirmed in clinical trials involving humans. If proven, this could pave the way for earlier diagnosis and faster initiation of therapy.

To fully understand the impact of the microbiome on the development of AD, large-scale population studies are needed to fully characterize the taxonomy and metabolic functions of microorganisms—not only bacteria, but also viruses, fungi, archaea, and protozoa. These lesser-known organisms may also influence inflammatory processes in the CNS and contribute to cognitive decline.

It is also worth noting that the gut and oral microbiota are only two components of the broader human microbiome. The microbiota of the respiratory system, skin, and urogenital tract remain insufficiently studied in terms of their impact on neurodegeneration—this aspect should also be analyzed in more detail.

The oral dysbiosis mentioned in the study may influence the development and progression of Alzheimer’s disease by causing inflammation in the brain. LPS and oral bacterial biofilm may promote the production and deposition of β-amyloid and enhance tau protein phosphorylation, thus playing a significant role in the pathogenesis of AD. However, further research is needed to fully understand the mechanisms and potential diagnostic or therapeutic applications.

Therapies based on antibiotics, prebiotics, and probiotics show promising results in animal models, improving cognitive function. Probiotics (e.g., Lactobacillus and Bifidobacterium) protect the brain by reducing inflammation and improving memory, while prebiotics (e.g., FOS, GOS, and XOS) support beneficial gut bacteria and improve synaptic plasticity in the CNS. In addition, diet also appears to be an important factor. The Mediterranean diet (MeDi), through its anti-inflammatory properties, may reduce the risk of AD, while the MIND diet (MeDi + DASH) may prove even more effective. Data on antibiotics, however, are inconclusive. Antibiotics reduce the diversity of the microbiota, which can lead to its destabilization and colonization by pathogens that promote neurodegenerative changes. On the other hand, studies in animal models show that broad and prolonged use of antibiotics may reduce amyloid β (Aβ) deposition, increase its soluble forms, and reduce glial activation in the brain. However, there is still a lack of well-designed clinical trials in humans to confirm the efficacy and safety of such interventions. Another promising direction is the personalization of therapy based on individual microbiota profiles. In the future, a modern therapeutic approach may combine classic anti-inflammatory and amyloid/tau-targeted drugs with modulation of the gut microbiome.

However, given the complexity of the mechanisms underlying Alzheimer’s disease, the question arises: will such therapy be sufficient? Most likely not. However, it can be an important supporting element in an integrated, multidirectional therapeutic approach. Only such comprehensive action can bring real results in the treatment of this difficult disease.

## Figures and Tables

**Table 1:** The summary information of the two main phyla of human intestinal microbiota is based on Section 3.2 [33,38,40,41,42,43,44,45,46] and Section 3.3 [29,33,38,39,40,41,42,43,44,48,49].

	Firmicutes	Bacteroidetes
**Basic construction**	Mostly Gram-positive with a thick peptidoglycan layer	Gram-negative cell wall structure, with an outer membrane built from (LPS).
**Diversity**	Over 200 genera, such as Ruminococcus, Clostridium, Eubacterium, Lactobacillus, Faecalibacterium, Roseburia, and Mycoplasma	Bacteria including the genera Bacteroides, Prevotella, and Xylanibacter produce enterotoxins that disrupt the intestinal barrier.
**Action of microbiota**	Fermentation of indigestible carbohydrates (such as dietary fiber) and the production of SCFAs; Responsible for better results in various types of tests, checking cognitive skills (GABA production)	Unclear function and importance of Aβ for the bacteria themselves and the host. Studies on this phylum have not so clear, either favorable or negative results.

**Table 2:** Summary of gut microbiota alterations in AD patients and animal models, indicating associated pro- or anti-inflammatory shifts and relevant references. This table is based on reports collected in recent years confirm the interactions taking place at the level of the gut–brain axis. The table focuses on the result of the interactions taking place, assessing the inflammatory effect of given strains on the axis, labeling them as pro-inflammatory (↑) or anti-inflammatory factors (↓). The references for dedicated works are given in the corresponding columns [38,39,44,51,52].

No.	Materials	Gut Microbiota	Main Effect	References
**1**	AD patients	*Firmicutes*	↓	[14]
**2**	AD patients	*Bacteroidetes*	↑/↓	[14,15,33]
**3**	AD patients	*Eubacterium rectale*	↓	[17]
**4**	AD patients	*Bifidobacterium*	↓	[14]
**5**	AD patients	*Actinobacteria*	↑	[15]
**6**	Transgenic mice	*Proteobacteria*	↑	[23]
**7**	AD patients	*Escherichia/Shigella*	↑	[17]
**8**	Transgenic mice	*Clostridium leptum*	↑	[24]
**9**	AD patients	*Lachnospiraceae*	↓	[15]

**Table 3:** Overview of the impact of antibiotics on the pathology and disease management in Alzheimer’s Disease. ↑—increase, ↓—decrease.

Antibiotic Class	Impact on AD & Microbiota	Study Type	References
**Broad-spectrum antibiotic therapy (cocktail)**	↓ deposited Aβ; ↑ soluble Aβ; attenuated plaque-localized glial reactivity; deep and long-lasting microbiota modification (depletion/remodeling)	Preclinical (mouse models)	[62]
**Tetracyclines (minocycline, doxycycline)**	Anti-aggregatory against Aβ/Tau; anti-inflammatory activity; potential microbiota modulation with long-term use	Preclinical (animal models)	[63,64]
**Rifamycins (rifampicin)**	Anti-aggregatory; ↑ hippocampal autophagy, ↓ Aβ; secondary effects on microbiota possible	Preclinical (animal models)	[65,66]
**β-lactams**	Signals of efficacy in slowing AD progression in preclinical studies; microbiota effects not specified in summary	Preclinical	[68]
**Quinolones**	Antibiotic-induced dysbiosis (altered SCFAs/LPS); possible ↑ AD risk	Cohort	[69]
**Sirolimus /Rapamycin**	TOR regulation; in animal models: restoration of hippocampus/caudate volumes and global CBF (APOE4 carriers); indirect effects on neurodegeneration pathways	Preclinical (animal models; short-term)	[13,67]

**Table 4:** Summary of potential biomarkers of AD based on gut microbiota. ↓—the level is lower in patients with AD, ↑—the level is higher in patients with AD.

Potential Biomarker	AD Patients Compared to Controls	References
**SCFA-producing bacteria (*Bacteroidaceae, Rikenellaceae*)**	↓	[70,71,72,73]
***Firmicutes, Bacteroidetes, Proteobacteriaceae, Verrucomicrobiaceae, Bifidobacteriaceae, Erysipelotrichaceae*** **, *Helicobacteraceae, Desulfovibrionaceae***	↑	[70,71,72,73]
**Level of Trimethylamine N-oxide (TMAO) in cerebrospinal fluid**	↑	[74]
**Short-chain fatty acids (SCFAs)**	↓	[75]
**Tryptophan metabolites**	↓	[75]
**Microbial diversity**	↓	[76]

**Table 5:** Key components of the Mediterranean diet and their benefits in the prevention of Alzheimer’s disease and neurodegeneration. The contents of the table are based on [86,87,88,89].

	Extra-Virgin Olive Oil (EVOO)	Soluble Fiber
**Main biological properties**	Rich in secondary phenolic compounds with strong antioxidant activity	Promotes beneficial gut microbiota composition
	Modulation of gut microbiota (increase in lactic acid bacteria)	Increases SCFAs production, especially butyrate
	Immunomodulatory effects (stimulation of the immune system: increase in mucosal IgA, decrease in plasma CRP- a systemic inflammatory marker)	Reduces propionate, linked with gut microbiota dysbiosis
	Anti-inflammatory action (reduction in inflammatory markers: MPO, 8-OHdG, TNF-α, IL-6)	Lowers astrocyte activation
	Increased adiponectin and regulation of IL-10 expression	
**Impact on the nervous system and cognitive functions**	Reduction in oxidative stress that damages neurons	Improves gut–brain communication via microbiota metabolites
	Support of immune homeostasis, lowering neuroinflammatory processes	Butyrate enhances neuroprotection and neuroplasticity
	Indirect support of synaptic functions via improved gut–brain axis	Reduced astrocyte activation supports neuronal integrity
**Protective effects in the context of Alzheimer’s disease**	Decreased risk of neurodegeneration caused by oxidative stress	Restoration of microbiota balance (prevention of dysbiosis)
	Reduction in brain inflammation	Improved cognitive performance (shown in AD animal models)
	Support of gut–brain axis through microbiota modulation	Reduction in neuroinflammatory and degenerative processes
	Overall slowing of neurodegenerative processes leading to AD	

## Data Availability

No new data were created or analyzed in this study.
